# Multidimensional diffusion MRI with spectrally modulated gradients reveals unprecedented microstructural detail

**DOI:** 10.1038/s41598-019-45235-7

**Published:** 2019-06-21

**Authors:** H. Lundell, M. Nilsson, T. B. Dyrby, G. J. M. Parker, P. L. Hubbard Cristinacce, F.-L. Zhou, D. Topgaard, S. Lasič

**Affiliations:** 1 Danish Research Centre for Magnetic Resonance, Centre for Functional and Diagnostic Imaging and Research, Copenhagen University Hospital Hvidovre, Hvidovre, Denmark; 2 Clinical Sciences Lund, Radiology, Lund University, Lund, Sweden; 3 Department of Applied Mathematics and Computer Science, Technical University of Denmark, Kongens Lyngby, Denmark; 4 Division of Neuroscience and Experimental Psychology, School of Biological Sciences, The University of Manchester, Manchester, M13 9PT United Kingdom; 5 Bioxydyn Limited, Manchester, United Kingdom; 6 Division of Physical Chemistry, Department of Chemistry, Lund University, Lund, Sweden; 7 Random Walk Imaging AB, Lund, Sweden

**Keywords:** Solution-state NMR, Biomedical engineering, Diagnostic markers

## Abstract

Characterization of porous media is essential in a wide range of biomedical and industrial applications. Microstructural features can be probed non-invasively by diffusion magnetic resonance imaging (dMRI). However, diffusion encoding in conventional dMRI may yield similar signatures for very different microstructures, which represents a significant limitation for disentangling individual microstructural features in heterogeneous materials. To solve this problem, we propose an augmented multidimensional diffusion encoding (MDE) framework, which unlocks a novel encoding dimension to assess time-dependent diffusion specific to structures with different microscopic anisotropies. Our approach relies on spectral analysis of complex but experimentally efficient MDE waveforms. Two independent contrasts to differentiate features such as cell shape and size can be generated directly by signal subtraction from only three types of measurements. Analytical calculations and simulations support our experimental observations. Proof-of-concept experiments were applied on samples with known and distinctly different microstructures. We further demonstrate substantially different contrasts in different tissue types of a post mortem brain. Our simultaneous assessment of restriction size and shape may be instrumental in studies of a wide range of porous materials, enable new insights into the microstructure of biological tissues or be of great value in diagnostics.

## Introduction

A wide variety of materials are porous in nature. Characterizing the microstructure of such materials is key to understand for example the state and function of biological tissues, elasto-mechanic properties of materials, or texture of food products and rocks. This range of porous materials can be non-invasively studied by diffusion magnetic resonance imaging (dMRI)^1^. However, despite the high sensitivity of dMRI, different microstructural features, such as size, shape and orientation of diffusion restricting pores or living cells may yield indistinguishable signatures in conventional dMRI^2–4^. In other words, different microstructural information may be entangled in the encoding process, leading to low specificity of conventional dMRI. This problem is particularly acute in tissue, which consists of diverse compartments residing within a typical millimeter-size imaging voxel.

The dMRI research community has traditionally tried to resolve different microstructural features of tissue indirectly by biophysical modeling^5^. However, due to the lack of clean, disentangled data providing orthogonal, independent information, this approach is prone to over-fitting leading to erroneous conclusions^6,7^. The problem of entangled information is also common in nuclear magnetic resonance (NMR) studies of solids and porous media, and the research community has learned to address this by multidimensional encoding approaches. It has been shown that the resolution power in investigations of heterogeneous materials can be significantly increased if the signal is encoded with several independent measurement dimensions simultaneously. This approach maximizes the specificity of data by preventing the entanglement at an encoding stage^8–12^.

One common challenge for dMRI is to resolve microscopic diffusion anisotropy from the macroscopic orientation of microscopic compartments. Conventional experiments can provide a unique signature related to pore shape, but only in very special circumstances, when a sample consists of monodispersed pores with identical shapes^13–15^. To detect microscopic anisotropy in heterogeneous systems, Cory *et al*. introduced diffusion-diffusion correlation NMR experiments in which displacements are encoded in two non-collinear directions^16^. This diffusion encoding approach, now known as double diffusion encoding, has recently been applied in a number of MRI studies^17^.

Under the assumption of Gaussian diffusion, the problem of resolving the distribution of diffusion tensors in systems with unknown macroscopic orientation dispersion, like for example in the brain tissue, is analogous to the problem of estimating distributions of chemical shift tensors for partially aligned polymer materials^8^. The solutions traditionally found in solid-state methodology, which can resolve isotropic and anisotropic nuclear interactions^18^, have recently inspired a more general multidimensional diffusion encoding (MDE) framework feasible both in NMR and MRI settings^19^. Prior approaches in this direction have extended the diffusion encoding from a vector-valued to a tensor-valued diffusion encoding^20,21^. The anisotropy of an encoding b-tensor, varying from sticks for directional encoding to spheres for isotropic encoding, is an example of the acquisition dimension, which matches the experimentally accessible microscopic diffusion anisotropy^22^. The general premise behind the MDE framework is to match the dimensionality of the acquisition space with that of the underlying phenomena^19^. Thus, it is crucial to identify relevant dimensions of the experimental inquiry and appropriately parameterize the acquisition space to create clean measurements of independent effects.

In this work, we consider non-Gaussian diffusion effects in MDE, which are related to time-dependent diffusivities and have traditionally been exploited with unidirectional encoding experiments to probe microstructure of porous media^23–26^. In complex systems, both time-dependent and anisotropic diffusion effects can be entangled, which may lead to biased results if only anisotropic diffusion is considered. Isolating those effects already at a measurement stage can thus provide more specific microstructural information. However, experimentally efficient sequences for MDE employ general encoding waveforms where the diffusion time is not well defined. Time-dependent diffusion can be analyzed in the frequency domain as the spectrum of velocity correlations^27^. This perspective enables experimental designs probing shorter length-scales using diffusion encodings with oscillating gradients^28–30^. As we demonstrate here, the frequency domain also provides an intuitive way to understand the effects of time-dependent diffusion across MDE protocols with more complex waveforms.

We suggest an augmented MDE protocol with specificity to both time-dependent diffusion and microscopic anisotropy. The observed effects relate to signal fractions with particular cell size and shape. Besides varying the shape of the encoding b-tensor so that the encoding is either isotropic or directional, we propose to independently vary also the encoding power spectrum as an additional encoding dimension. Importantly, varying the encoding power spectrum provides the necessary mechanism to match time-dependent diffusion effects in isotropic and directional encodings, which employ experimentally effective but complex encoding waveforms. We have applied directional encoding, which was either “tuned” or “detuned” with respect to the spectra of isotropic encoding. Our experimental design and analysis provide two important and valuable insights. First, the tuned directional encoding is needed for an unbiased detection of microscopic anisotropy when time-dependent diffusion effects are prominent over the length-scales probed by MDE waveforms. Second, our protocol provides additional and independent information revealing correlations between anisotropy and size of structures on the micrometer length scale. These two independent and highly specific contrasts are attainable directly by raw image subtraction. We have demonstrated that our method can provide a clear separation of distinctively different synthetic and biological microstructures. We anticipate that our method will be of high value in applications, where a prompt assessment of heterogeneous microstructure is required as for example in MRI assessments of pathologies.

## Theory

### Directional and isotropic diffusion encoding

We consider previously introduced optimized gradient waveforms for isotropic encoding based on the magic-angle spinning of the q-vector (q-MAS)^31^. For Gaussian diffusion, the isotropic encoding is orientation invariant and sensitive only to the isotropic diffusivity in each component. The observed multi-exponential signal decay is in this case, in contrast to the directional encoding, only affected by the dispersion of isotropic diffusivities^22^. The average microscopic anisotropy can thus be inferred from the difference between signals acquired with directional and isotropic encoding in a material with a uniform orientation distribution of microstructures. A uniform distribution can be mimicked in any material by averaging the signals acquired in multiple gradient orientations, i.e. by so called *powder averaging*^22,32,33^.

### Time-dependent diffusion

**Figure 1:**
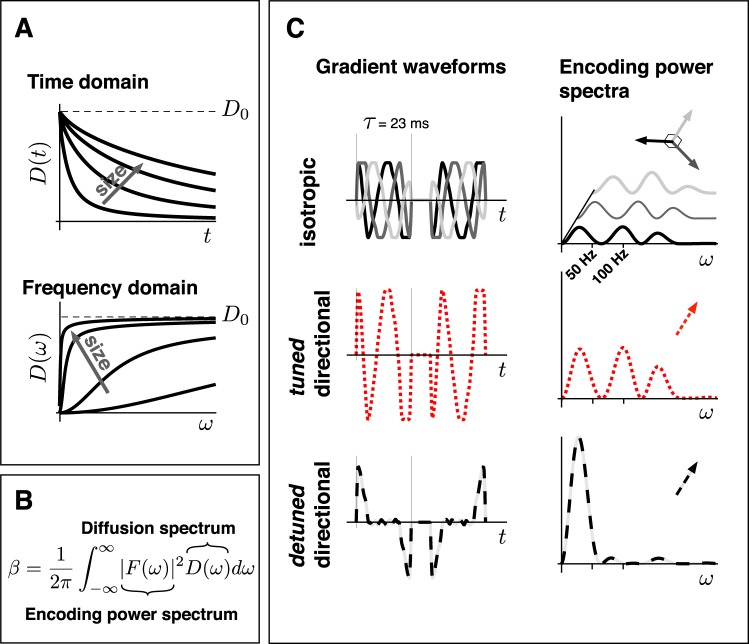
Experimental design considerations for time-dependent diffusion. (**A**) Time-dependent diffusion can be considered in either time or frequency domains. For general diffusion encoding gradient waveforms, the diffusion times are ill defined. Thus, the frequency domain formulation provides a more stringent description of the signal. (**B**) The signal attenuation factor *β* from an individual compartment is given by the diffusion spectrum filtered by the encoding power spectrum. (**C**) The gradient waveforms (left panels) and the corresponding encoding power spectra (right panels) used in this study. The isotropic encoding has similar encoding power spectra in three orthogonal directions (different shades of gray) (top). The two directional encodings have the same total encoding power, i.e. *b*-value, but with either similar (tuned) or different (detuned) power spectra compared to the isotropic encoding. Note the increased encoding power at lower frequencies in case of detuned encoding. Time and frequency scales are shown for our experimental settings with the encoding time *τ* = 23 ms.

Note that even in the limit of low diffusion weighting *b*-values, where the signal attenuation from a single compartment can be considered approximately mono-exponential and spin-phase dispersion due to diffusion approximately Gaussian, a multi-exponential decay is observed for a system with multiple polydispersed restrictions or molecules with different diffusivities (free diffusion)^34^. In the literature, any multi-exponential signal attenuation has often been attributed rather vaguely to “non-Gaussian diffusion”. We describe diffusion as “time-dependent” to distinguish it from the case of free diffusion even when it applies to a multi-Gaussian scenario. Time-dependent diffusion *D(t)* can be viewed in the time domain, but for experiments with finite encoding times the diffusion time is ill defined and the diffusion spectrum *D*(*ω*) proves to be a more convenient concept (Fig. 1A). The signal attenuation in the Gaussian approximation of cumulant expansion^34^ is then given by the sum of all the spectral components of *D*(*ω*) weighted by the encoding power spectrum ${|F(\omega )|}^{2}$, which depends on the gradient waveform^27^ (Fig. 1B,C). See methods section for details. Note that the diffusion weighting *b*-value is given by a sum of all the spectral components of ${|F(\omega )|}^{2}$.

Time-dependent diffusion will affect the results of an MDE experiment when there is a significant variation of the diffusion spectrum *D*(*ω*) in the frequency range where the encoding power, according to ${|F(\omega )|}^{2}$, is present. In this regime, the inferred anisotropy will depend on the spectral profile of the applied MDE gradient waveforms.

### Augmented multidimensional diffusion encoding protocol

In our new experimental protocol, we combine the isotropic encoding with two different directional encoding waveforms shown in Fig. 1C to detect a non-flat *D*(ω). We call them tuned and detuned. The tuned waveform has a similar encoding power spectrum, |*F*(ω)|^2^, as the isotropic encoding waveform and will thus measure the same apparent diffusion coefficient (ADC) in an isotropic sample. For the detuned waveform we use the originally suggested directional encoding^31^. In our experiments, both directional and isotropic encoding yield identical *b-*values, but the detuned directional encoding features considerably more power at lower frequencies compared to the tuned version, as can be seen in Fig. 1C. In a multi-compartment system, different sizes of restrictions can be measured based on the difference in signals from the tuned and detuned encodings, while anisotropy differences can be measured by comparing the tuned directional and isotropic encodings.

## Results

The augmented MDE protocol was implemented on a 4.7 T preclinical MRI system and experiments were conducted on four phantoms with well-known microstructures as well as on a post mortem monkey brain tissue. All data shown represent powder averaged signals. For quantification of the average microscopic fractional anisotropy (µFA) and the degree of macroscopic alignment of anisotropic compartments, we refer to the earlier work^22^. Further methodological details are provided in the method section.

### Stratification of anisotropy and size in porous phantoms

**Figure 2:**
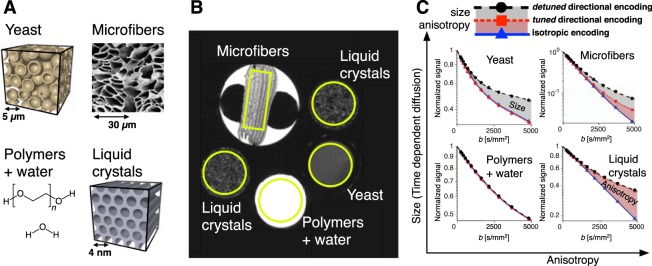
Experiments on various porous materials. (**A**) Illustrations of the four phantoms used in the experiment: yeasts cells represent large isotropic restrictions, microfibers large anisotropic restrictions, liquid crystals small anisotropic restrictions and polymer solution exhibits free diffusion. Data from all phantoms where collected in the same imaging experiment. (**B**) *T*_2_-weighted image of the four phantoms. Signals from the individual phantoms were averaged over regions indicated by the yellow lines. (**C**) Powder averaged and normalized signals for three encoding waveforms shown in Fig. 1C. The phantoms are schematically ordered according to the degree of anisotropy and size. The divergent signals from detuned and tuned encodings reveal time-dependent diffusion in the larger structures (gray shading in yeast and microfibers). The difference between signals for the tuned directional and isotropic encodings reveals anisotropy (red shading in liquid crystals and microfibers). For a mixed system, the signal differences are proportional to the signal fractions of the individual components.

To demonstrate the effects of time-dependent and anisotropic diffusion probed by our augmented MDE protocol, we constructed an array of diffusion phantoms with four distinctively different microstructures (Fig. 2A). The phantom data were collected within one imaging session (Fig. 2B). The effects of anisotropy and size were evaluated as signal differences at different *b*-values (Fig. 2C).

#### Aqueous polymer solution

The polymer solution phantom contains polymers (polyethylene glycol) dissolved in water^35^. All three signals from the polymer solution overlap, which is consistent with time-independent and isotropic diffusion (lower left Fig. 2C). The multi-exponential signal decay reflects multi-Gaussian diffusion, related to the different spin populations in the solution (free self-diffusion of water and polymers of different lengths).

#### Yeast cell suspension

The densely packed baker’s yeast cell suspension, with spherical cells of ~5 µm diameter, features both extracellular and intracellular compartments, with hindered (time-independent) and restricted (time-dependent) diffusion, respectively^22^. The isotropic diffusion in both compartments is reflected in the multi-exponential signal decays, which overlap for the tuned directional and isotropic encoding. However, time-dependent diffusion in the intracellular compartment causes a divergence of the tuned and detuned directionally encoded signals (upper left Fig. 2C).

#### Liquid crystals

The liquid crystals phantom comprises orientationally dispersed anisotropic domains having an internal structure with ~4 nm diameter aqueous channels on a 2D hexagonal lattice with ~7 nm repeat distance^20^. The signal acquired on liquid crystals manifest the opposite behavior compared to the yeast results. The tuned and detuned directional encodings have their respective encoding power below ~200 and 50 Hz (Fig. 1C) and in this case yield overlapping results. Considerably higher frequencies would be required to detect any water mobility perpendicular to the channels (lower right Fig. 2C). In contrast to the signals from directional encoding, the isotropic encoding yields a nearly mono-exponential signal decay, suggesting that the directional signal attenuation is fully explained by identical but orientationally disordered domains in liquid crystals with apparently 1D anisotropic and time-independent diffusion.

#### Microfibers

This phantom comprises liquid filled hollow polymeric microfibers with inner diameters around 10 µm^36^. The water channel diameters of the hollow fibers are three orders of magnitude larger than for the liquid crystals. In this case, ${|F(\omega )|}^{2}$ for the tuned directional encoding has sufficient power in the range of frequencies where variation of $D(\omega )$ is present, which is manifested in the difference between the results for the tuned and detuned directional encoding (gray shaded area in the upper right Fig. 2C). Just like in case of liquid crystals, the signal attenuation from isotropic and tuned directional encodings diverge due to anisotropy (red shaded area in the upper right Fig. 2C). The multi-exponential decay of the isotropic encoding indicates a residual dispersion of apparent isotropic diffusivities due a various fiber diameters, unrestricted water compartments within the fibers phantom or variations in the spectral content across different directions of the isotropic encoding^36,37^.

### Examples of biological tissue

**Figure 3:**
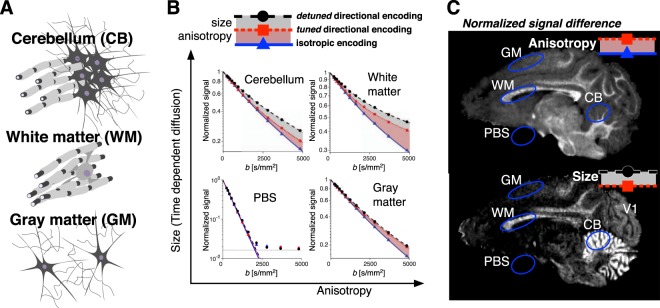
Experiments on post mortem monkey brain. (**A**) Schematic illustration of the different tissue compositions contained within three regions of interest (ROIs). The cerebellum (CB) contains white matter, dendrites and in particular a very high density of neuronal cell bodies in the inner granular layer of the cerebellar cortex. White matter (WM) contains mainly myelinated axons and a small proportion of glial cells. Gray matter (GM) contains mainly dendrites and neuronal and glial cell bodies. (**B**) Powder averaged and normalized signals for the three encoding waveforms shown in Fig. 1C. Similarly as in Fig. 2C, the results from the four ROIs are ordered according to the degree of anisotropy and size. (**C**) Maps reflecting anisotropy (upper) and cell size (lower) calculated from the powder averaged signal differences at *b* = 4800 s/mm^2^ voxel-wise normalized to the *b* = 0 signal. The contrasts correspond to the signal differences shown in panel B as gray and red shaded areas. The primary visual cortex (V1) has slightly higher intensity in the size map compared to the gray matter in the rest of the cerebrum.

A more complex example of heterogeneous microstructures, commonly studied by dMRI, are biological tissues. We performed the same experiments on an *ex vivo* excised Vervet monkey brain^38^, where three distinctly different microenvironments, schematically shown in Fig. 3A, are expected. The corresponding signals are shown in Fig. 3B.

The cerebellar region of interest (ROI) consists of a mixture of axons and dendrites as well as the granular layer with a very high concentration of spherical neuronal cell bodies (Fig. 3A). This region exhibit both a signal difference between the tuned directional and isotropic encoding from anisotropy (red shaded area in Fig. 3B) as well as a substantial effect of time-dependent diffusion, as inferred from the tuned and detuned directional encoding (gray shaded area in Fig. 3B).

White matter consists of aligned axons with anisotropy both in the intra- and extracellular compartments (Fig. 3A). The signal difference between the tuned directional and isotropic encodings is maximal compared to other regions (red shaded area in Fig. 3B). Effects of time-dependent diffusion inferred from the signal difference between the tuned and detuned directional encoding is also pronounced but smaller compared to the cerebellum (gray shaded area in Fig. 3B).

In gray matter, water diffusion may occur in anisotropic dendrites or a more isotropic extracellular space (Fig. 3A). In our experimental data, the anisotropy in gray matter is reflected by the signal difference between the tuned directional and the isotropic encoding (red shaded area in Fig. 3B), while only a very small signal difference between the tuned and detuned directional encoding (gray shaded area) indicates that time-dependent diffusion is not pronounced.

The phosphate buffer ROI (PBS) serves as a control region and is located outside the neuronal tissue. It is chemically and structurally homogeneous on the micrometer length scale probed by dMRI. Consistent with the expected Gaussian diffusion, all three signals overlap and are mono-exponential above the rectified noise floor (lower left Fig. 3B).

The signal differences at the highest *b*-value shown in Fig. 3B are in the order of 5–15%, which allows voxel-wise visualization of the contrasts as shown in Fig. 3C. The strong time-dependent diffusion contrast is visible mainly in the cerebellar cortex and white matter. Interestingly, the pattern of low time-dependent diffusion contrast in gray matter is broken in the primary visual cortex (V1 in. Fig. 3C), featuring intermediate time-dependent diffusion contrast.

### Insights from calculations and Monte Carlo simulations

**Figure 4:**
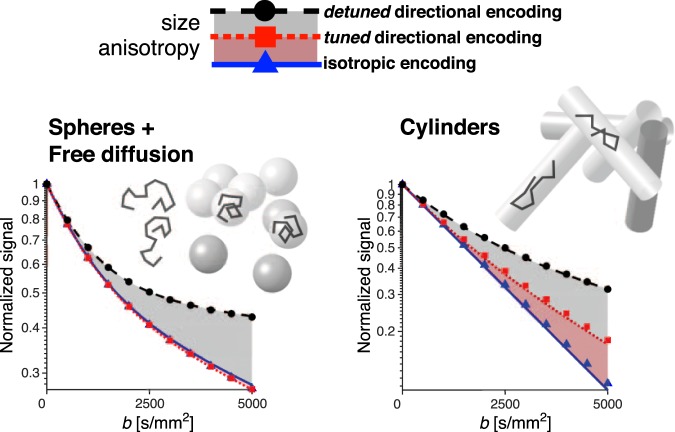
Signals calculated from the analytical description (lines) and Monte Carlo simulations (markers) for the system with impermeable spheres embedded in a compartment with free diffusion (left) and for the system with uniformly oriented impermeable infinite cylinders (right). The signal vs. *b* curves were calculated for the two systems with restriction radius *R* = 2.5 µm and for the encoding time *τ* = 23 ms. The same *τ* was used in our experiments (Fig. 1) yielding results shown in Figs 2C and 3C.

To provide a model-based interpretation of our encoding contrasts, we performed calculations and Monte Carlo simulations. Effects of restricted diffusion in our augmented MDE protocol were considered for a broad range of encoding times and restriction sizes for two types of systems: (1) an isotropic system consisting of two compartments, one with Gaussian diffusion and one with diffusion restricted within identical impermeable spheres, each compartment contributing 50% of the signal, (2) a macroscopically isotropic but microscopically anisotropic system consisting of identical impermeable cylinders with uniform orientation distribution. Figure 4 shows signal vs. *b* curves for spherical and cylindrical restrictions with dimensions and experimental parameters comparable to those in our experiments on yeast and microfiber phantoms. The theoretical predictions in Fig. 4 qualitatively correspond with the experimental results shown in Fig. 2C. For diffusion inside a restriction, the time-dependent diffusion contrast, i.e. the difference between the tuned and detuned encoding, depends on the relation between the frequency content of the two waveforms and the restriction dimensions. Further considerations on those relations are presented in the Supplementary Information.

## Discussion

In this paper we propose an MDE experimental approach that is specific both to microscopic anisotropy and time-dependent diffusion by the use of three custom designed gradient waveforms. The use of b-tensor encoding on its own has been demonstrated successfully in both NMR and MRI including experiments on liquid crystals^39^ and the histologically validated differentiation of cell morphology in different brain tumor types^40^. On the other hand, time-dependent diffusion, traditionally studied with directional encoding schemes, was reported early on in frog muscle^30^ and has later been extensively investigated in porous media and in different biological tissues^2,24,29,41–43^. Human *in vivo* examples of time-dependent diffusion observations include various tumor types^44,45^ and the healthy and ischemic human brain^46,47^. Our approach could eliminate ambiguities when combined effects of various degrees of microscopic anisotropy and time-dependent diffusion may interfere for instance in different types of tumor tissue. *Tuning* of the frequency profiles in MDE allows for a clean separation of these effects and provides additional independent microstructural information.

In this work we demonstrated an MDE application with data driven specificity related to size and shape of microstructures. Our method addresses the limitation of conventional dMRI techniques, which are highly sensitive but not adequately specific since information about size and anisotropy is entangled, yielding indistinguishable signal attenuation signatures. We show how more specific microstructural information can be retrieved if, in addition to varying the encoding b*-*tensor shape, also the sensitivity to time-dependent diffusion is varied within the MDE framework. Our framework thus allows relating apparent anisotropic diffusivities to structural length-scales.

Frequency-domain analysis provides an intuitive understanding of how MDE waveforms encode time-dependent diffusion. Based on this, we suggest an augmented MDE protocol with spectrally modulated gradient waveforms. Spectral *tuning* ensures that the effects of time-dependent diffusion are kept constant while varying the encoding b*-*tensor shape, which is key for accurate assessment of anisotropy in heterogeneous anisotropic porous media such as biological tissues. Most importantly, spectrally modulated MDE provides an additional independent encoding dimension, which allows sorting microstructures according to the degree of anisotropy and time-dependent diffusion.

Our proof of concept experiment on phantoms featuring distinctly different microstructures illustrates the problem we have addressed and demonstrates the potential of our method. The attenuation curves for the detuned directional encoding, which alone resembles a conventional dMRI experiment, shows vaguely multi-exponential signal attenuations in all examples. Inferring underlying microstructure in those materials would require multiple severe *a priori* model assumptions. The three measurements with different b-tensor shapes and encoding power spectra but the same diffusion weighting *b*-value can clearly separate the four phantoms featuring microstructures with different size and anisotropy. Phantom data supported by simulations exemplify the clean measurement dimensions. Time-dependent but isotropic diffusion is identified by a distinctly different signal for the detuned encoding, while the signals for the tuned and isotropic encoding are identical (yeast data in Fig. 2 and spheres in Fig. 4). In contrast, anisotropic diffusion in absence of time-dependent diffusion effects is identified by a distinctly different signal for the isotropic encoding, while both tuned and detuned directional encodings yield identical signals in this case (liquid crystals in Fig. 2 and thin tubes corresponding to long relative encoding times in Fig. 5, see also Supplementary Information).

The detuned directional encoding waveform used in our experiments resembles encoding with two short gradient pulses, where the effective diffusion time is approximately equal to the encoding time $\,\tau$. In the special case of pulsed field gradient encoding, the relation between apparent diffusivity and restriction size is well known^48–50^. Sensitivity to restriction size, which to the first order depends on apparent diffusivity, can be inferred also for arbitrary encoding waveforms based on the spectral analysis. As we show in Fig. 5 (Supplementary Information), maximum sensitivity occurs when the characteristic displacement of freely diffusing molecules during the encoding time, $\sqrt{{D}_{0}\tau }$, approximately matches restriction size R. In this regime, differences between the signal attenuations for the tuned and detuned encodings are expected to be maximal. Our analytical and simulated results (Supplementary Information) can thus be used to guide interpretation of our experimental observations.

Neuronal and glial cell bodies are typically in the order of 3–10 µm in diameter, which is within the sensitivity range of our encoding parameters (see Supplementary Information). Most axons, dendrites and other cellular structures are below 1 µm but with a broad size distribution^51^. The time-dependent diffusion in white matter could occur perpendicular to axons due to the restrictions of axonal membranes with radii above several micrometers or due to extracellular space featuring structural heterogeneities over length scales of 10–100 µm^52^. Time-dependent diffusion may also be caused by intra-axonal varicosities acting as polydisperse semipermeable barriers spaced along axons in the range of 5–10 µm or due to axonal undulations^47,53^. The observed effect of time-dependent diffusion in regions with densely packed cell bodies, e.g. in the cerebellar granular layer (“size” contrast in Fig. 3C), is well known from earlier *post mortem* studies using oscillating gradient spin echo techniques applying encoding power in a similar range of frequencies around 100 Hz and is validated with histology^29,54^. The intermediate time-dependent diffusion contrast observed in the primary visual cortex (V1 in. Fig. 3C) is consistent with the sharp increase of neuronal cell body density by a factor of 2–3, which is known to exist in this region of gray matter^55,56^. The larger anisotropy observed in white matter compared to gray matter could be attributed to a larger fiber density or to a slower exchange of water between the extra- and intracellular spaces in myelinated axons compared to dendrites^57^.

In MDE, different encoding axes may have different encoding power spectra, which means that in the presence of anisotropic time-dependent diffusion even a spherical encoding b*-*tensor, i.e. isotropic encoding, does not yield orientationally invariant signal attenuation^37^. In analogy to the anisotropy of the encoding b*-*tensor, MDE waveforms possess an additional property of relevance that we call spectral anisotropy. Varying spectral anisotropy may provide yet another valuable independent dimension in MDE, which is exclusively sensitive to time-dependence in anisotropic compartments^58^. For an isotropic encoding under powder average conditions, as applied in this paper, the average power spectrum needs to be considered. To achieve proper spectral *tuning* between directional and isotropic encodings, one should ideally find a rotation of the isotropic encoding axes, which yields identical power spectra along the orthogonal encoding axes matching the average power spectrum. This may not be attainable exactly; however, as we demonstrated, the differences between the power spectra along the orthogonal axes of our q-MAS implementation are sufficiently small that an efficient *tuning* between the isotropic and directional encoding can be achieved. The effect of spectral anisotropy in our isotropic encoding is readily observed based on theoretical predictions and Monte Carlo simulations (highlighted region in Fig. 5D of Supplementary Information). However, this effect is negligible in comparison to the signal differences between isotropic and tuned/detuned directional encoding.

A conceptually simpler approach to study time-dependent and anisotropic diffusion could be based on applying single or repeated blocks of short gradient pulses or oscillating gradients with alternating gradient directions and well-defined diffusion times^59–61^. However, such experiments may not always be feasible, in particular in practical imaging settings. Gradient hardware performance in terms of maximum gradient strength, slew rate and duty cycle as well as the relaxation properties of the sample limit the experimentally accessible parameter space. Using smoother gradient waveforms makes it possible to access MDE parameters within a measurable range^31^. In our case, neither the encoding time nor a single encoding frequency can adequately characterize gradient waveforms. It is rather a distribution in frequency content of the entire encoding that provides a useful link between experiments and observations^62^.

Previous 2D experiments have probed multi-Gaussian time-independent diffusion by dense sampling of points spanning a full range of axisymmetric b-tensors^20,39^ or with additional dimensions sampling directions^63^. Dense 2D sampling schemes have also been done for time-dependent diffusion with correlations between diffusivity sampled at two different diffusion times^11^. In contrast, sparse sampling of a limited but most informative multidimensional data is a good strategy when time and hardware limitations need to be considered. Our experiments confirm that very rich information can be obtained when acquisition dimensions are carefully designed despite our minimal sampling protocol with just three data points. Along the same idea, we have previously proposed sparse sampling schemes to isolate local self-diffusion from exchange and incoherent flow effects^64,65^. Future extended dense sampling protocols could control time-dependent diffusion effects by keeping the tuning constant when varying tensor shapes or probe time-dependent diffusion by varying encoding power spectrum along an independent third encoding dimension. A trivial way of varying the encoding power spectrum in practice is achieved by simply varying the encoding time, as done in our simulations (Supplementary Information Fig. 5). The echo-time should in such experiments be constant or varied independently to control for or explore entangled relaxation effects^66,67^. Finally, in our analysis we rely on the Gaussian approximation of the signal cumulant expansion, i.e. assuming that the signal attenuation from each compartment is mono-exponential. This assumption is reasonable at low values of diffusion weighting^34^. In our Monte Carlo simulation, we found that deviations from mono-exponential decay in an individual cylindrical restriction prior to powder averaging, indicating the breakdown of the Gaussian approximation, were less than 1% for the isotropic encoding in the range of *b*-values and encoding times used in this study.

As Douglas Adams notoriously illustrated by 42 as the “Answer to the Ultimate Question of Life, the Universe, and Everything”^68^, the value of the *ultimate answer* intrinsically depends on the value of the *ultimate question*. In line with this parable, we advocate diffusion encoding designs, which can ease the requirements for model assumptions and fitting involved in explaining experimental observations. As our results demonstrate, we can facilitate interpretations by providing more *direct contrasts* specifically sensitive to time-dependent anisotropic diffusion in heterogeneous media. Importantly, considering the magnitude of our contrasts in the raw signal is crucial before seeking too elaborate answers through modeling and potential over-fitting. Our experimental results, supported by theoretical predictions and simulations, clearly demonstrate the ability of multidimensional diffusion encoding to directly resolve qualitatively different microstructural properties. Future applications of our method include differentiation of heterogeneous tumor tissue and other diagnostics where prompt and non-invasive assessments of changes in cell morphology are essential.

## Materials and Methods

### Model systems and tissue preparation

The phantoms were prepared as follows. The aqueous polymer solution was prepared with 50 wt% polyethylene glycol with molecular weights between 1305 and 1595 g/mol (Sigma Aldrich, MO, United States) dissolved in H_2_O and filled in a 10 mm glass tube. Fresh baker’s yeast was mixed with water in 1/1 weight ratio. The cell suspension was left overnight to sediment. Residual water was removed, and the yeast sediment was transferred to a 10 mm glass tube and centrifuged at 2500 g for 10 minutes to produce a solid sediment of yeast cells without CO_2_ bubbles. The top water layer was again removed, and the tube was sealed. The polydomain lyotropic liquid crystals of the reversed hexagonal type were prepared as previously described and filled in two 10 mm glass tubes^20^. A system with larger diameter tubular restrictions was realized with cyclohexane filled microfibers with a mean diameter of 13.4 µm^36^. An excised brain from a 3.5 year old vervet monkey (*Chlorocebus sabeus*) was prepared for *ex vivo* imaging^38^. The post mortem monkey brain specimen was obtained from the Behavioral Science Foundation, St. Kitts and the live animal was socially housed in enriched environments. All procedures for handling and sacrificing the live animal prior to our post mortem experiment were reviewed and approved by the Institutional Review Board of the Behavioral Science Foundation acting under the auspices of the Canadian Council on Animal Care. No experimental procedures were performed on the live animal as a part of this study. The whole brain was submerged in minimal amount of phosphate buffer solution (PBS) in a plastic bag. Care was taken to release air bubbles trapped in the tissue before the bag was sealed.

### MRI

Data were acquired on a 4.7 T preclinical MRI system with a 600 mT/m gradient set (Agilent Technologies, Santa Clara, CA, USA). The tubes with the phantoms where bundled together and placed horizontally in a 30 mm quadrature transmit/receive coil (Rapid MR International, Columbus, OH, USA). Care was taken to ensure stable temperature at different gradient loads. A small animal monitoring system was used to stabilize the temperature during different gradient loads. A heated air flow through the scanner bore was controlled by the temperature measurement from a probe attached to the inner housing of the coil just outside the imaging field of view (SA Instruments Inc., Stony Brooke, NY, USA). The temperature was logged and kept at 23.0 ± 0.2 °C throughout the whole experiment. The diffusion protocol was run for 2 hours prior to data acquisition to achieve a stable working temperature and to minimize short-term instabilities due to physical handling^38^.

Maximum gradient strength of 500 mT/m was applied using an optimized isotropic encoding^31^. The spectrally tuned directional encoding provided by the x-channel of the isotropic encoding scheme was scaled by $\sqrt{3}$ to match the same *b*-value. A spectrally detuned directional encoding using the gradient waveform with dephasing corresponding to the dephasing magnitude of the isotropic encoding was applied as in previous studies^22^. Since the gradient waveforms were played out symmetrically around the refocusing pulse, no detrimental effects of concomitant fields were expected^69^. Experiments were repeated with 15 uniformly distributed orientations of the gradient axes and with 12 *b*-values logarithmically spaced between 240 and 4800 s/mm^2^. The gradient waveforms with encoding time *τ* = 23 ms were performed before and after the refocusing pulse in a spin-echo sequence. Images were acquired with a 2D single line readout using an echo time of 68 ms and a repetition time of 2500 ms.

All imaging parameters were identical for the phantoms and for the post mortem monkey brain experiment except for the imaging matrix and resolution. In the phantom experiment, the imaging matrix was 128 × 128 × 3 and the voxel size 0.375 × 0.375 × 3 mm^3^. A single image without diffusion encoding with a matrix size of 256 × 256 × 3 and voxel size of 0.18 × 0.18 × 3 mm^3^ was acquired and used for visualization in Fig. 2. For the post mortem brain experiment, the imaging matrix was 128 × 256 × 5 (number of phase encoding steps was kept constant) with a voxel size of 0.25 × 0.25 × 2 mm^3^. Total imaging time was 48 hours for each experiment.

### Experimental data analysis

Reconstructed images were exported from the scanner and analyzed using in house code written in Matlab (Mathworks Inc., Navick, MA, USA). Signal intensities were powder averaged across measurements with the same b-tensor shape and *b*-value. The signal attenuation was fitted to 3rd order in *b* using nonlinear least squares fit to the signals with the *b* = 0 signal as an additional free parameter to plot the signal vs. *b*-value curves in Figs 2 and 3. Regions of interest (ROIs) where manually drawn on the images to extract the signals from the different phantoms and from the different tissue types in the monkey brain. Subtraction maps shown in Fig. 3C were calculated after normalization to the estimated unweighted image and smoothed with an isotropic Gaussian kernel with 5 mm full width at half maximum.

### Analytical calculations

Under Gaussian approximation of the signal cumulant expansion^34^, the effects of time-dependent diffusion in arbitrary diffusion encoding waveforms can be analyzed in frequency domain^27,29^. Ignoring relaxation effects, normalized signal attenuation is given by, *E* = exp(−*β*) where the attenuation factor *β* is given by the diffusion spectra **D**(*ω*) as^27^

1 $$\beta =\frac{1}{2\pi }{\int }_{-\infty }^{\infty }{{\bf{F}}}^{{\rm{T}}}(\omega ){\bf{D}}(\omega ){\bf{F}}(-\omega )d\omega .$$

Here **F**(*ω*) is the spectrum of the dephasing vector **F**(*t*),

2 $${\bf{F}}(\omega )={\int }_{0}^{\tau }{\bf{F}}(t){e}^{-i\omega t}dt,$$

given by the effective gradient waveform **g**(*t*) as

3 $${\bf{F}}(t)=\gamma \,{\int }_{0}^{t}{\bf{g}}(t^{\prime} )dt^{\prime} ,$$

where *γ* is the gyromagnetic ratio. The dephasing spectrum along direction $\hat{{\bf{n}}}$ is given by ${F}_{\hat{{\bf{n}}}}(\omega )={\bf{F}}(\omega )\hat{{\bf{n}}}$ and the corresponding power spectrum is given by ${|{F}_{\hat{{\bf{n}}}}(\omega )|}^{2}$. We use the term encoding power spectrum as a synonym for the dephasing power spectrum. **D**(ω) can be expressed with a rotation matrix **R** as **D**(ω) = **Rλ**(ω)**R**^−1^, where **λ**(ω) is the diagonal matrix containing diffusion spectra *λ*_*i*_(*ω*) along the restriction principal axes. Analytical expressions for the *λ*_*i*_(*ω*) in case of restricted diffusion in cylindrical, planar and spherical geometries can be found in the literature^27^. The total diffusion encoding power, more commonly known as the *b-*value, is given by the trace of the b*-*tensor,

4 $${\bf{b}}={\int }_{-\infty }^{\infty }{\bf{F}}(\omega )\otimes {\bf{F}}(\,-\,\omega )d\omega =\,{\int }_{0}^{\infty }{\bf{F}}(t)\otimes {\bf{F}}(t)dt,$$

where ⊗ denotes outer product and the equivalence between the frequency and time domain formulations is a consequence of Parseval’s theorem. The apparent diffusion coefficient is proportional to the initial slope of the 1n(*E*) vs *b* curve and is defined as ADC =$\,\beta /b$.

The dephasing spectra ${\bf{F}}(\omega )$ were calculated with the FFT algorithm (Mathworks Inc., Navick, MA, USA) using a time step of 1 µs and 125·10^4^ points with varying amount of zero padding depending on the encoding time $\tau$. Calculations were performed for $\tau$ between 50 µs and 625 ms, the intrinsic diffusivity *D*_0_ = 1·10^−9^ m^2^/s and spherical and cylindrical compartments of various sizes (*R* = 1, 2.5, 5, 10 µm). Results only depend on the relative encoding time $\sqrt{{D}_{0}\tau }/R$ (support data not shown). Signal attenuation, i.e. signal vs. *b* curves for the case of *τ* = 23 ms and *R* = 2.5 µm are shown in Fig. 4, and the corresponding mean ADC values and their variances are shown in Fig. 5 (Supplementary Information) for a range of relative encoding times.

ADC = *β/b* values were computed for each out of 1000 uniformly distributed orientations according to Eq. 1. Spherical restrictions were considered in a mixture with a Gaussian diffusion compartment with diffusivity *D*_0_ contributing 50% of the signal. The mean ADC value over different orientations, 〈ADC〉, and its variance, *V*_D_ = 〈[ADC − 〈ADC〉]^2^〉, were computed over a wide range of relative encoding times. 〈ADC〉 and *V*_D_ respectively reflect the initial slope and curvature of the signal vs. *b* curves, given by exp[−〈ADC〉*b* + *V*_D_*b*^2^/2] (see Supplementary Information Fig. 5A,C).

### Monte carlo simulations

Monte Carlo simulations were performed as a cross-validation of the frequency domain analysis using an algorithm written in Matlab (Mathworks Inc., Navick, MA, USA). The simulation was performed with spherical and cylindrical restrictions with radii of 2.5 µm and gradient waveforms identical to those used in the frequency domain analysis. Gradient amplitudes were adjusted to achieve *b*-values in the range 0–5000 s/mm^2^ for all encoding times. The intrinsic diffusivity was set to *D*_0_ = 1·10^−9^ m^2^/s. The random walk process was performed with 5000 time steps and 10^5^ diffusing particles. Restrictions were modeled by rejecting steps crossing barriers^70^. The simulation was repeated with the symmetry axis of the substrate rotated in 60 uniformly distributed directions relative to the gradient frame of reference. The ADCs for each gradient waveform and orientation were computed from the exponential decay between two lowest *b*-values (0 and 100 s/mm^2^). The 〈ADC〉 and *V*_D_ values shown in Fig. 5A,B (Supplementary Information) were computed as for the analytical results.

## Supplementary information

Supplementary information

## Data Availability

The datasets generated and analyzed during the current study are available from the corresponding author on reasonable request.
