# Supplementary material for: Multidimensional diffusion MRI with spectrally modulated gradients reveals unprecedented microstructural detail
Source: Sci Rep. 2019 Jun 21;9:9026. doi: 10.1038/s41598-019-45235-7 (PMC6588609; doi:10.1038/s41598-019-45235-7)
Supplement: Supplementary file 1 — Supplementary information [file 41598_2019_45235_MOESM1_ESM.pdf]

SUPPLEMENTARY INFORMATION

Multidimensional diffusion MRI with spectrally modulated gradients reveals unprecedented microstructural detail

H. Lundell[1*], M. Nilsson[2], T.B. Dyrby[1,3], G.J.M Parker[4,5], P.L. Hubbard Cristinacce[4], F.-L. Zhou[4], D. Topgaard[6] and S. Lasič[1,7]

1. Danish Research Centre for Magnetic Resonance, Centre for Functional and Diagnostic Imaging and Research, Copenhagen University Hospital Hvidovre, Hvidovre, Denmark,
2. Clinical Sciences Lund, Radiology, Lund University, Lund, Sweden,
3. Department of Applied Mathematics and Computer Science, Technical University of Denmark, Kongens Lyngby, Denmark,
4. Division of Neuroscience and Experimental Psychology, School of Medical Sciences, The University of Manchester, Manchester M13 9PT, United Kingdom,
5. Bioxydyn Limited, Manchester, United Kingdom,
6. Division of Physical Chemistry, Department of Chemistry, Lund University, Lund, Sweden,
7. Random Walk Imaging AB, Lund, Sweden

*Corresponding author:
Henrik Lundell

Address:
Danish Research Centre for Magnetic Resonance
Copenhagen University Hospital Hvidovre
Kettegaards Allé 30
3480 Hvidovre
Denmark
E-mail: lundell@drcmr.dk
Phone: +45 38620505

ADDITIONAL THEORETICAL CALCULATIONS AND SIMULATIONS

The sensitivity to size inferred from the difference between the tuned and detuned encodings depend on the relation between restriction size, intrinsic diffusivity and encoding time. As a universal scaling, we introduce the relative encoding time $\sqrt{D_0\tau}/R$ where D_0 is the intrinsic diffusion coefficient unaffected by the barriers, τ is the encoding time of the gradient waveform (here defined as the length of each block repeated twice around the refocusing pulse) and R is the restriction size. Figs. 5A and 5B show the mean apparent diffusivity, ⟨ADC⟩, demonstrating that the effects of time-dependent diffusion (difference between the dashed black and solid blue lines) is maximal in an intermediate regime where $\sqrt{D_0\tau}/R \sim 1$. For shorter relative encoding times, the apparent diffusivity approaches D_0, while at long times, the apparent diffusivity approaches the tortuosity limit, which is zero in the case of restricted diffusion. The diffusion variance, V_D, shown in Figs. 5C and 5D, is proportional to the deviation from mono-exponential decay to second order in b also known as kurtosis[1]. It reflects the differences between apparent diffusivities in a multi-compartment system. For all encoding schemes, the time-dependent variances approach asymptotic values at short and long relative encoding times[2]. The diffusion variance in Fig. 5C stems from the difference between the Gaussian diffusion component with high apparent diffusivity and the restricted diffusion component with lower apparent diffusivity. The larger encoding power present at lower frequencies in the detuned directional diffusion encoding yields lower apparent diffusivities and consequently larger V_D compared to the tuned directional and isotropic encodings. The same effect comes into play with cylindrical restrictions (Fig. 5D), but here it is reflected only in the difference between the apparent diffusivities parallel and orthogonal to the main cylinder axis.

Note that for a system composed of a single anisotropic compartment with Gaussian diffusion, isotropic encoding always yields $V_D = 0$. The same result is expected also in case of time-dependent diffusion if the encoding power spectra along all projections of the isotropic encoding are perfectly matching. However, when the encoding is isotropic only in terms of the b-tensor and not in terms of the encoding power spectra, the rotational invariance may be compromised in systems with time-dependent anisotropic diffusion, resulting in a residual V_D as noted by de Swiet and Mitra using time domain analysis[3]. This relatively small effect can be seen as the residual variance for the isotropic encoding in the highlighted region in Fig. 5D.

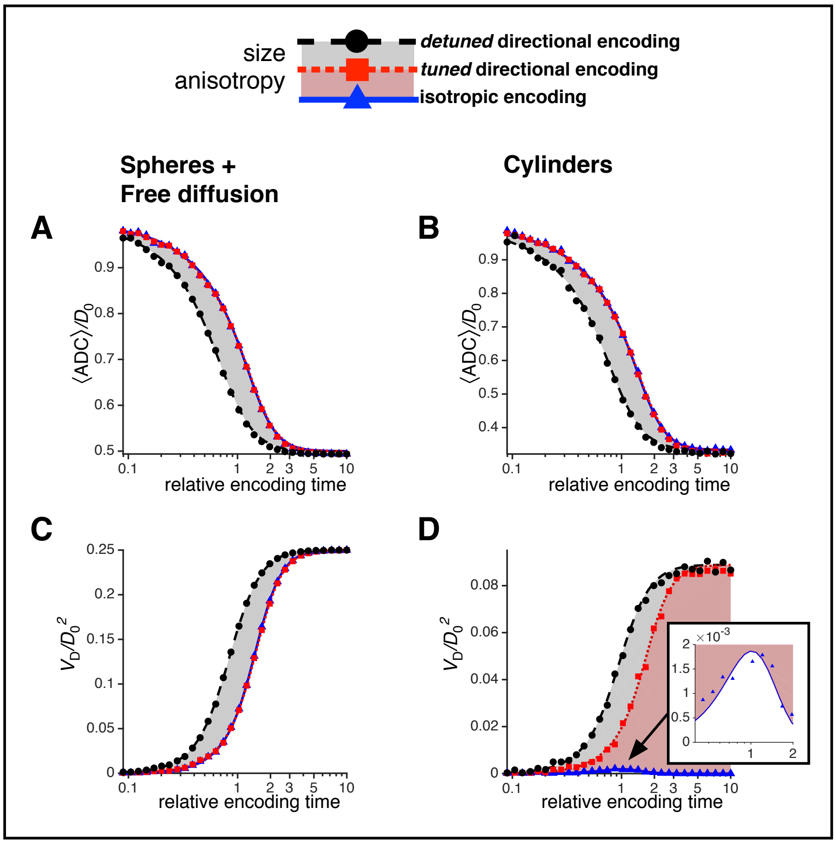

Legend (Figure 5): The effect of varying encoding time and restriction size. Results of analytical predictions (lines) and Monte Carlo simulations (markers) over a range of relative encoding times, $\sqrt{D_0\tau}/R$, given by the ratio between the characteristic displacement of freely diffusing molecules during the encoding time τ and the restriction size R. Two systems were considered: 1) impermeable spheres embedded in a compartment with free diffusion (A, C) and 2) impermeable infinitely long cylinders (B, D). Panels A and B show the scaled powder averaged diffusivity $\langle\mathrm{ADC}\rangle/D_0$ over a range of relative encoding times. The average diffusivity reflects the initial slope of attenuation curves like those shown in Figs. 2C, 3B and 4. Panels C and D show the corresponding variance in ADC values, V_D, proportional to the b^2-term in the cumulant expansion of the signal. The arrow in panel D highlights the non-zero V_D in the case of cylinders and isotropic diffusion encoding, which is attributed to the directional variation of the encoding power spectrum.

References

1. Jensen, J. H., Helpern, J. A., Ramani, A., Lu, H. & Kaczynski, K. Diffusional kurtosis imaging: The quantification of non-Gaussian water diffusion by means of magnetic resonance imaging. *Magn. Reson. Med.* **53,** 1432–1440 (2005).
2. Nilsson, M., Lasič, S., Drobnjak, I., Topgaard, D. & Westin, C.-F. Resolution limit of cylinder diameter estimation by diffusion MRI: The impact of gradient waveform and orientation dispersion. *NMR Biomed.* **30,** e3711 (2017).
3. de Swiet, T. M. & Mitra, P. P. Possible systematic errors in single-shot measurements of the trace of the diffusion tensor. *J. Magn. Reson. - Ser. B* **111,** 15–22 (1996).
